# Building Effective and Equitable Global Midwifery Collaborations: Research, Education, and Clinical Learning

**DOI:** 10.1111/jmwh.70039

**Published:** 2025-10-30

**Authors:** Michelle Telfer, Rachel Zaslow, Sande Ojara, Joan Combellick, Scovia Nalugo Mbalinda

**Affiliations:** ^1^ Yale School of Nursing West Haven Connecticut; ^2^ Mother Health International Courtesy Faculty Yale School of Nursing Atiak Uganda; ^3^ Department Obstetrics and Gynecology St. Mary's Hospital Lacor‐Gulu University Teaching Hospital Gulu Uganda; ^4^ Department of Nursing College of Health Sciences Makerere University Kampala Uganda

**Keywords:** birth centers, community health, global health, interprofessional collaboration, international, midwifery education, midwifery models of care

## Abstract

The historically unidirectional movement of global health ideas, practices, and protocols from the Global North (United States, Canada, European countries, Japan, South Korea, Taiwan, Australia, New Zealand, and Israel) to the Global South (Latin American countries, African countries, the Middle East excluding Israel and Asia countries, and Oceania excluding those previously mentioned) has displaced local practice and produced little sustainable change. Actively addressing these unintended consequences, practitioners at Yale School of Nursing in the United States formed a sustainable, mutually beneficial partnership with Makerere University College of Health Sciences and Mother Health International community birth center in Atiak, Uganda, to reduce perinatal mortality in areas with the highest burden. Goals included establishing a collaborative midwifery education and research partnership; developing an interprofessional clinical rotation; and developing a blueprint for teaching the midwifery model of care in the Global South. The partnership has successfully produced outputs including midwifery education support, research, clinical training, interprofessional capacity building, and community integration within local health care systems. Lessons learned from program design, implementation, and evaluation can inform global learning collaborations that are multidirectional and lead to more equitable midwifery collaborations.

## INTRODUCTION

Rooted in colonial medicine, the field of global health continues to prioritize Western knowledge and experience.^1^, ^2^ Scientific institutions in high‐income countries (HICs) privilege Western thought as the global standard. Attempts to bring low and middle‐income countries (LMICs) into the conversation are often limited and require members to adhere to standards set by the HICs. The terms *Global North* and *Global South* came into use to move away from pejorative terms such as first‐, second‐, and third‐world countries and the “developing” countries system. Although not perfect, the North‐South terminology focuses on the international power and prosperity of countries. Communities in the Global South often struggle to adhere to practices that are not readily applicable to their unique setting for reasons ranging from unsupported supply chains to a lack of cultural appropriateness or context.^2^, ^3^, ^4^, ^5^ Shortages of health care professionals in the Global South are made worse by the flow of trained health professionals to more lucrative jobs in the Global North.^6^ As a result, countries with the highest disease burden often cannot respond rapidly or effectively to their community's health care needs.

A current manifestation of the historic health care imbalances centers on the exploding desire of health care students in the Global North for clinical experiences in the Southern regions. Students seek international experiences for personal enrichment or to feel they are contributing to a better world, sometimes with religious intent.^4^ Institutions have responded by offering short‐term global health experiences, or STEGH.^4^, ^7^ Currently, 99% of American medical schools offer STEGH, and more than one‐quarter of students participate.^7^ Nursing and midwifery schools justify STEGH to prepare graduates to work with diverse patient populations.^8^ Unfortunately, few programs consider the substantial impacts on local communities.^9^, ^10^

  

Continuing education (CE) is available for this article. To obtain CE online, please visit http://www.jmwhce.org. A CE form that includes the test questions is available in the print edition of this issue.

Host countries report power dynamic differentials with visitors from HICs, with participants feeling disrespected and discriminated against.^7^ The host country's limited resources may be strained and local students displaced during clinical rotations. Overworked host staff must assume the burden of supervising HIC learners who have little clinical training.^9^ With limited accountability and oversight, students pose a significant threat to individuals receiving care. Although funding has been poured into STEGHs, little financial aid goes to local health systems, infrastructure, or education.^7^

**QUICK POINTS**

✦ Global health historically has replicated a neocolonial, unidirectional model of knowledge transfer from the Global North that has caused harm and disruption to the Global South.
✦ North‐to‐South partnerships based on co‐created goals and following an ethical framework and guidelines can benefit and empower all partners.
✦ Improving global perinatal outcomes will require the teaching, supporting, and replication of midwifery models of care.

Cross‐cultural collaborations between health institutions risk power imbalances, unidirectional benefits, and potential harm. In response, Crump et al^11^ published guidelines outlining best practices for training experiences in global health. In 2018, the American College of Nurse‐Midwives (ACNM) created the Global Health Competencies and Skills to provide educational and clinical guidance to US‐based midwives in the global context.^12^ The Brocher Declaration^13^ is the most recent effort to formalize an ethical framework for global engagement. Signed by universities, organizations, and governments, it recognizes the current and past harm perpetrated by unregulated STEGH and outlines principles to ensure ethical engagement and improve the impacts on host communities.^13^ These documents underpin the collaboration described in this article (see Table 1).

**Table 1:** Collaboration Work and Outputs Alongside American College of Nurse‐Midwives Global Health Competencies and Skills and The Brocher Declaration

ACNM Global Health Competencies	Brocher Declaration Guidelines	Collaborative Work and Outputs
Global understanding	Mutual partnership with bidirectional input and learning	Virtual 15‐wk global midwifery seminar with master's midwifery students and faculty from both universities and nonprofit organization (syllabus developed based on QMNC framework); 12 students, 4 faculty Coursera course based on the QMNC framework with 14,000 students enrolled over 5 y (course content: Maternal and Newborn Health Metrics, Quality Maternal and Newborn Care & the Midwifery Model of Care, Care Delivery, Care Organization, Care Values, Care Philosophy, Care Providers, Policy and Advocacy) 15‐wk predeparture seminar for HIC students prior to their immersive clinical experience based on the ACNM GHC (39 students enrolled over 5 y) Virtual Midwifery Grand Rounds: midwifery students from both universities present case studies from their practice during a synchronous online session.
Clinical practice		Skills laboratory for HIC students: Helping Babies Breathe, Helping Mothers Survive, Low‐Resource Settings Skills transfer from Ugandan midwives, obstetrician‐gynecologists, and traditional midwives to HIC faculty and students during immersions Skills transfer from HIC university faculty to Ugandan midwives, traditional midwives, and obstetrician‐gynecologists Uganda students’ immersion at birth center: learning from traditional midwives
Health equity and justice	Empowered host country and community define needs and activities	Health equity and justice is centered in the 3 courses developed and curricula for community immersion for all learners Host country drives agenda: skills, research, capacity building workshops Centering the community and paying host country traditional midwives, midwives, and interpreters for precepting students
Professionalism/ethics	Compliance with applicable laws, ethical standards, and code of conduct	Midwives/MDs from HIC apply for and attain licenses in the host country and follow the national guidelines and ministry of health recommendations No learner practices or performs skills outside their scope of practice or experience at home
Communication	Humility, cultural sensitivity, and respect for all involved	All courses/curricula emphasize cultural humility, respectful care and respect for other ways of knowing, and Indigenous knowledge Integration with traditional midwives respecting Indigenous knowledge and skills
Leadership, organization, and program management	Accountability for actions	Primary collaborators from all partners have equal input and respectful communication
Teaching/learning	Mutual partnership with bidirectional input and learning	Multiple courses, curricula and webinars co‐designed, co‐created, and co‐produced All partners have presented to faculty, students and colleagues in both settings
Research/continuous quality improvement	Sustainable programs and capacity	Multiple grant submissions Articles published presentations/poster at regional, national, and international conferences by all partners
Health system strengthening		Capacity building with training, curricula, mentorship, tools package at other facilities, and development of a train the trainers program Partner with Ministry of Health and spread program across Northern Uganda, Uganda, and East Africa

Abbreviations: ACNM, American College of Nurse‐Midwives; GHC, Global Health Competencies; HIC, high‐income country; MD, doctor of medicine; QMNC, Quality Newborn Maternal Care.

### Midwifery and Perinatal Health in Global Context

The global health community has long advocated for health facility–based birth and a scale‐up of skilled birth attendants to reduce preventable perinatal mortality. Although licensed midwives are critical contributors to strengthening health systems and addressing health care demands as described in the Sustainable Development Goals,^14^, ^15^ midwifery professionalization has devalued and sidelined traditional midwifery and traditional birth attendants, risking the loss of critical cultural knowledge and diminishing community trust.^16^, ^17^ Many licensed midwives view traditional midwives negatively and refuse to help when a woman is transferred to a health facility.^17^ There are limited examples of the two working together to provide culturally relevant care.

*The Lancet's* 2014 series on midwifery^18^ highlighted that cultural knowledge and community trust are critical to reducing unnecessary mortality in childbirth. The series’ Quality Newborn Maternal Care (QMNC) framework^19^ provides guidelines on averting more than 80% of such deaths by implementing the midwifery model of care.^20^ Accessible, respectful, and culturally appropriate care is critical to this framework's success in improving outcomes.^19^

As the push for licensed midwives grows, many countries struggle to meet the demand, and midwifery educational programs fall short of the International Confederation of Midwives (ICM) essential competencies.^21^ Focus on clinical skills often displaces care that is based in trust and respect intrinsic to midwifery. In Uganda, certificate midwives comprise close to 90% of practicing midwives, yet they receive only 6 weeks of maternal health–specific training.^22^, ^23^ There is no midwifery model of care component in the national Ugandan curriculum.^23^, ^24^ The World Health Organization's recent global position paper^25^ on Transitioning to Midwifery Models of Care defines midwifery models of care as models in which educated autonomous midwives are the main providers through pregnancy, birth, and postpartum. Midwives coordinate respectful, person‐centered care across health care settings; optimize physiological, biological, psychological and cultural processes; and judiciously use interventions.^25^ No standards or training replaced the cultural and person‐centered care that was stripped away with the eradication of traditional midwifery.^26^ These gaps must be addressed to implement the midwifery model of care and improve outcomes.^27^

## EVOLUTION OF A COLLABORATION

In 2006, medical faculty at Makerere University (MU) in Uganda and Yale University (YU) in the United States created a partnership, now known as MUYU. Co‐directed by physicians at MU School of Medicine and YU School of Medicine, this collaboration is built on bidirectional exchanges, learning, and research. In 2017, the midwifery departments from both universities were invited to join MUYU. Initial meetings held in Uganda led to the development of shared partnership goals centered on midwifery education, research, and clinical exchanges. Key faculty stakeholders spent 2 years designing their partnership to develop mutual trust and understanding before they introduced student rotations. They developed a robust framework that was flexible and centered the needs of the community. To prepare highly skilled midwives able to offer high‐quality, culturally competent care, the MUYU team designed a site‐based learning module at a rural birth center employing the midwifery model of care. This collaboration has engendered complex and strong professional and personal relationships among the MUYU faculty, an essential component of a sustained collaboration.

The midwifery shortage in Uganda^22^ necessitated a deep‐rooted partnership based on equitable practice. Systems‐level change requires a broad, immersive, and slow approach to understanding community needs and midwife education and training. The collaboration grew to include Ot Nywal Me Kuc (House of Birth and Peace) community birth center in Northern Uganda, founded by Mother Health International (MHI) in 2008. MHI's model embraces traditional midwives (called community health workers) who work alongside licensed midwives. Despite rural poverty, distance from high‐level facilities, and a population recovering from war, the birth center, working closely with the Ministry of Health and the community, has outcomes that mirror those in the Global North.^28^ The birth center provided the perfect site for in‐depth and meaningful course development and clinical immersions for students from both contexts.

### Strengthening Midwifery Education

MU's masters in midwifery was the first master's midwifery program to open in East Africa. The program was due for reaccreditation in 2018, and MU faculty had that curriculum as well as a new and first‐of‐its‐kind masters in pediatric nursing curriculum reviewed by YU faculty. The universities convened a national stakeholder meeting to introduce the pediatric curriculum and provide support for its implementation.

MUYU midwifery faculty created a 15‐week online seminar based on the QMNC framework. Jointly taught, this asynchronous and synchronous hybrid course involved weekly online guided discussions and activities. Students participated in high‐level learning about global midwifery while creating cross‐cultural connections with future colleagues. Course evaluations and reflections demonstrated high satisfaction levels.

MUYU midwifery faculty capitalized on the success of this course to launch a massive online open course: Global Quality Maternal and Newborn Care on the Coursera online educational platform.^29^ Designed around the QMNC Framework^19^ and focused on global maternal and newborn care, the course sought to bring broader knowledge about midwifery and the midwifery model of care to health professionals, policy makers, government stakeholders, community stakeholders, and consumers from the Global North and South^29^ (See Table 2). The course has reached more than 15,000 participants and has received highly positive reviews.

**Table 2:** Coursera Global Quality Maternal and Newborn Care Modules

**Maternal and Newborn Health**	**Care Organization**	**Care Philosophy**
Sustainable Development Goals Maternal Mortality Maternal Mortality in the United States Global Stillbirth Stillbirth Child Survival: Neonates & Children Under 5	High‐Quality, Low Cost, Collaborative Care The Role of Midwifery Cambodia Case Study Integrative Midwifery Care: Rural Northern Uganda Integrating Midwives in the United States Gender Inequality Africa Case Study Care Organization: Network Integration (aka Networks of Care)	Care Philosophy: What Matters to Women Emerging Science: Epigenetics and Birth Interventions at Birth Interventions at Birth and the Infant Microbiome Supporting Biological Processes and Implementing Change Uninterrupted Skin‐to‐skin Contact Building Capacity through Positive Birth Experience Care Philosophy: Creating Positive Birth Experiences

**Quality Maternal and Newborn Care & the Midwifery Model of Care**	**Care Values**	**Policy and Advocacy**
Quality Maternal & Newborn Care: The Lancet Series on Midwifery What is a midwife? Midwifery Model of Care: Philosophy Midwifery‐Led Models of Care The Expanded View of Midwifery Midwifery Care as Justice Work	Meeting the Needs of Individuals: Respect Identifying Disrespect at the Provider Addressing Disrespect at the System‐Level Meeting the Needs of Providers: Barriers Meeting the Needs of Families and Communities: Culture Culturally appropriate Training Examples Care Values: Women‐Centered Action Plans	Evidence in Practice: Our Approach to Policy Evidence in Practice: Midwives & Policy Advocacy Spotlight: UNFPA Advocacy Spotlight: White Ribbon Alliance

**Care Delivery**	**Care Providers**	
Too Little Too Late & Too Much Too Soon Quality Care: Practice Categories Evidence‐Based Care Guidelines Birth Setting The Built Birth Environment Birth Setting & Empowerment	Care Providers: Enabling the Workforce Scaling Up Midwifery Care: Impact and Cost Building Midwifery Workforce in India Building Midwifery Workforce in Africa Strengthening Quality Midwifery Education	

Source: https://www.coursera.org/teach/global‐quality‐maternal‐and‐newborn‐care/course/overview.

MUYU faculty then collaborated on designing a course to prepare YU midwifery students for clinical immersion at the MHI birth center. The curriculum focuses on filling gaps in knowledge, centering community voices, and working with traditional midwives to ensure continuity of care. YU midwifery students attend a semester‐long seminar to prepare for birth center immersion. Course objectives follow the ACNM Global Health Competencies and Skills^12^ and center the history of health colonization, ethics, cultural humility, and respect for indigenous knowledge. Students learn about working in low‐resource settings, trauma‐informed care, locally available medications, and endemic diseases. MUYU faculty prepare students to respectfully learn about the day‐to‐day lives of the women they care for alongside the Ugandan licensed and traditional midwives. Birth center midwives and traditional midwives receive a stipend for their precepting YU students.

MUYU midwifery faculty also collaborated on designing a Community Midwifery course for the MU master's students to prepare them for community integration and to practice the midwifery model of care. The course addresses known gaps in the Ugandan education system around critical organization of care in the Ugandan context. It concludes with a month‐long immersion at the birth center where students are first exposed to the midwifery model of care and given hands‐on training and mentorship in collaborative care.

The MUYU collaboration continued virtually through the COVID‐19 pandemic. MU faculty requested YU midwifery faculty to provide a series of webinars focused on simulation in health sciences education. With support from YU simulation department the webinars reached universities across East Africa. YU faculty provided 14 webinars on implementing simulation into midwifery, nursing, medicine, and health sciences education. The webinars received excellent evaluations and reached hundreds of health care professionals.

MUYU faculty are beginning to co‐create a hybrid online course for midwifery and obstetrician‐gynecologist trainees about to embark on their community rotations to the birth center and nearby referral hospital. This course will foster understanding and collaboration across high‐resource and low‐resource settings as well as across professions. MUYU faculty and trainees will co‐create the curriculum with community members and traditional midwives to ensure its applicability.

### Student Clinical Immersions

Seeking to model multidirectional collaboration between midwives, community health workers, and physicians practicing in many settings, the partnership has co‐created curricula, didactic courses, and clinical experiences for midwives, learners, and obstetrician‐gynecologists from the United States and Uganda that reject the voluntourism model. The MHI birth center, where traditional midwives work collaboratively with licensed midwives, elevates traditional midwives as the keepers of cultural knowledge and community trust while ensuring rigorous, evidence‐based clinical care from licensed midwives. Participants develop a better understanding of why collaborative, nonhierarchical midwifery models of care have led to positive results for high‐risk populations.^30^

YU midwifery students live on the birth center compound for 4 weeks, attending births, participating in outreach care, preparing and cooking meals, and spending time with staff and their families. Students learn continuity of care and work with a local interpreter so all patients receive care in their own language. Two YU School of Nursing faculty accompany the students to support cultural assimilation and emotional processing so the Ugandan staff are not burdened. Daily debriefing sessions help students process and contextualize care. Skills workshops teach local remedies, homeopathy, and management of obstetric emergencies in low‐resource settings.

MU midwifery students spend a month at the birth center learning midwifery community health. This is unique in Uganda, where few licensed midwives interact with traditional midwives. Students participate in collaborative antenatal outreach with licensed and traditional midwives and learn how to implement responsive continuity of care. Faculty from both universities provide workshops on elements missing in the Ugandan midwifery curriculum, such as supporting physiologic birth with movement during labor, upright birth, provision of privacy, respectful care, and community engagement.

Evaluations are conducted with faculty, traditional and licensed midwives, and learners after each rotation via solo and group interviews, reflections and anonymous surveys. Students complete course evaluations and prepare project papers or presentations that are shared with collaborators. Partners use feedback and evaluations to improve the immersions after each rotation.

MUYU obstetrician‐gynecologist trainees have rotations at the MHI birth center and local referral hospital, St. Mary's Lacor, about an hour away by ambulance. This interprofessional collaboration links health care services along a continuum. Obstetricians‐gynecologist trainees learn to implement a midwifery model of care alongside traditional and nurse midwives and experience immersive community health integration. In turn, they provide training on topics requested by birth center staff such as vacuum‐assisted birth and management of miscarriage. MUYU faculty are working to strengthen these rotations by overlapping them with midwifery students and increasing interprofessional collaboration. The preimmersion course previously described is one feature of this. Both faculty and learners are eager to work and learn together in this unique setting. Three sets of YU obstetrician‐gynecology faculty and residents and one set from MU have rotated at the birth center and St. Mary's Lacor hospital and reported positive experiences, as have staff, and the collaboration plans to expand this rotation more formally in the coming year.

## DISCUSSION

This collaboration sought to understand the histories of problematic collaborations and sought guidance on creating an ethical partnership. In wanting to offer clinical rotations to learners, MUYU created a strong collaborative framework grounded in respect for local culture and care, and not as a stand‐alone opportunity that is unidirectional. This included establishing nonhierarchical shared‐decision‐making about research, training, education, and funding goals. Teams must be prepared for many iterations of shared goals and methodologies for research, funding, grant stewardship, teaching, and authorship.

When done well, the benefits of global experiences for trainees in reciprocal contexts are numerous. Deep understanding of the midwifery model of care makes new midwives and obstetrician‐gynecologists uniquely prepared to work in many settings and with diverse patient populations.

Faculty benefit from academic exchanges with mutually agreed‐upon research collaborations. Midwifery education, the midwifery model of care, and the QMNC framework are primary research interests of the collaborating faculty. One study^27^ examined the pathways and curricula for all midwifery programs in Uganda against ICM standards. A second^28^ focused on understanding the remarkable outcomes at the birth center alongside the QMNC framework.

## Implications For Research And Practice

### Research

The MUYU collaboration is expanding access to evidence, research, and the benefits of midwifery globally. MUYU faculty have presented at multiple national and international conferences on their work and partnership and have submitted more than a dozen grant applications over the past 7 years. Partners continue to pursue funding for research, quality improvement projects, and implementation science.

### Expanding the Model

Over 7 years, MUYU has produced outcomes that align with the ACNM Global Health Competencies^12^ as well as the Brocher Declaration^13^ (see Table 1). Partners plan to expand the MHI midwifery model through collaboration with nearby health centers and hospitals using a targeted curriculum, strong longitudinal mentorship, and key clinical resources and tools. In collaboration with the Ministry of Health and local hospitals, they will pilot a health facility transformation near the birth center in 2025‐2027 to scale up midwifery education collaboration. New partners will have opportunities to work alongside one another and share cross‐cultural ideas and practices. MUYU is working with the Ministry of Health to secure funding and ongoing institutional support toward expanding the program throughout Northern Uganda.

## Limitations And Challenges

As universities from the Global North seek to meet the demands for global experiences, there must be a funding mechanism that supports faculty in developing deeper collaborations and a framework for students to enter global experiences that reduce the burden and harm often caused by STEGH. Collaborations depend on grant funding for research, education, and quality improvement, but the ability to continue a partnership longitudinally also requires steadfast personal and institutional support.

As with any academic partnership, challenges arise with changes in faculty or leadership. Administrative changes at both universities have impacted the collaboration. The ongoing engagement of key faculty provided the necessary continuity to move the program forward. Maintaining this kind of deep‐rooted and longitudinal relationship is time intensive. Faculty may not be credit loaded appropriately for the work needed to support grant writing, student coordination, and travel logistics. Additionally, faculty who travel with students for clinical immersion must be able to leave other work and personal obligations for extended periods.

A key limitation to date has been the inability for the Yale School of Nursing to bring MU midwifery faculty to the United States due to financial, pandemic, and political challenges. The MUYU program has a grant that has supported dozens of MU medical faculty and medical students over the past 19 years for observer rotations at Yale. Grants are currently being pursued to support Makerere and MHI midwifery faculty to visit Yale.

## CONCLUSION

For those in the Global North wanting to engage in a cross global collaboration, it is important that groundwork is done to understand historical issues and potential harm that have occurred to those with whom they seek to partner. An ethical framework of equity, justice, and bidirectional reciprocity is critical. Cross‐cultural collaborative education, research, and clinical practice promise robust impact throughout the global health sector but require ongoing commitment from individuals and institutions. Meaningful collaborations are not established in a short period of time, but rather nurtured over years, establishing trust through transparent sharing and commitment to work through inevitable differences. This example of a collaboration embodies evidence‐based best practices, highlights the power of intercultural relationships, and supports interprofessional collaborations. The authors hope that these experiences and model may serve other institutions, educators, and clinicians who plan to work together across cultures and settings.

## CONFLICT OF INTEREST

The authors have no conflicts of interest to disclose.
